# Putting health facilities on the map: a renewed call to create geolocated, comprehensive, updated, openly licensed dataset of health facilities in sub-Saharan African countries

**DOI:** 10.1186/s12916-025-04023-z

**Published:** 2025-04-07

**Authors:** Peter M. Macharia, Lenka Beňová, Nicolas Ray, Aline Semaan, Moses M. Musau, John Kapoi Kipterer, Mark Herringer, Robert W. Snow, Emelda A. Okiro

**Affiliations:** 1 Department of Public Health, Institute of Tropical Medicine, Antwerp, Belgium; 2 Population and Health Impact Surveillance Group, Kenya Medical Research Institute-Wellcome Trust Research Programme, Nairobi, Kenya; 3 Faculty of Epidemiology and Population Health, London School of Hygiene & Tropical Medicine, London, UK; 4 Geohealth Group, Institute of Global Health, Faculty of Medicine, University of Geneva, Geneva, Switzerland; 5 Institute for Environmental Sciences, University of Geneva, Geneva, Switzerland; 6 World Health Organization Regional Office for Africa, Brazzaville, Congo; 7 The Global Healthsites Mapping Project, Hoorn, The Netherlands; 8 Nuffield Department of Medicine, Centre for Tropical Medicine and Global Health, University of Oxford, Oxford, UK

**Keywords:** Health facility database, Sub-Saharan Africa, Data, Healthcare, Health system, Open data sharing, Pandemics, Disease surveillance

## Abstract

**Background:**

Healthcare service provision, planning, and management depend on the availability of a geolocated, up-to-date, comprehensive health facility database (HFDB) to adequately meet a population’s healthcare needs. HFDBs are an integral component of national health system infrastructure forming the basis of efficient health service delivery, planning, surveillance, and ensuring equitable resource distribution, response to epidemics and outbreaks, as well as for research. Despite the value of HFDBs, their availability remains a challenge in sub-Saharan Africa (SSA). Many SSA countries face challenges in creating a HFDB; existing facility lists are incomplete, lack geographical coordinates, or contain outdated information on facility designation, service availability, or capacity. Even in countries with a HFDB, it is often not available open-access to health system stakeholders. Consequently, multiple national and subnational parallel efforts attempt to construct HFDBs, resulting in duplication and lack of governmental input, use, and validation.

**Main body:**

In this paper, we advocate for a harmonized SSA-wide HFDB. To achieve this, we elaborate on the steps required and challenges to overcome. We provide an overview of the minimum attributes of a HFDB and discuss past and current efforts to collate HFDBs at the country and regional (SSA) levels. We contend that a complete HFDB should include administrative units, geographic coordinates of facilities, attributes of service availability and capacity, facilities from both public and private sectors, be updated regularly, and be available to health system stakeholders through an open access policy. We provide historical and recent examples while looking at key issues and challenges, such as privacy, legitimacy, resources, and leadership, which must be considered to achieve such HFDBs.

**Conclusion:**

A harmonized HFDB for all SSA countries will facilitate efficient healthcare planning and service provision. A continental, cross-border effort will further support planning during natural disasters, conflicts, and migration. This is only achievable if there is a regional commitment from countries and health system stakeholders to open data sharing. This SSA-wide HFDB should be a government-led initiative with contributions from all stakeholders, ensuring no one is left behind in the pursuit of improved health service provision and universal health coverage.

**Supplementary Information:**

The online version contains supplementary material available at 10.1186/s12916-025-04023-z.

## Background

### What is a health facility database?

Healthcare service provision, planning, and management, with a view to adequately meeting a population’s healthcare needs, all depend on the availability of a geolocated, up-to-date, comprehensive health facility list that contains information on capacity and services provided at each health facility [1]. Such a list, henceforth referred to as a health facility database (HFDB) is also commonly referred to as a Master Facility List (MFL) or Health Facility Master List (HFML) [1]. These terms have been used interchangeably with a facility registry service; however, in this article, HFDB refers to the actual list of health facilities and associated data, whereas facility registry service refers to the software or tool that houses the HFDB allowing interoperability with other data systems [1]. Specifically, HFDB is a database of all health facilities in a country or area, containing—at a minimum—their unique identification (ID), name, facility level/designation, ownership, operational status, geographic location, and services endorsed (officially approved or designated) [1]. Such an authoritative list should be accessible to stakeholders including government authorities, implementing and development partners, and researchers through a facility registry service.

### What is the value and the benefit of a HFDB?

A HFDB constitutes an integral component of the national health system infrastructure. It is essential for planning and sustaining health services. The benefits of a HFDB in routine programs are large and can be seen at different levels (individual, community, sub-national, country, and continent). At the individual level, it offers a choice of facilities with valuable information on service availability and location. At the community level, it will keep health facilities accountable and facilitate the optimization of community services. For example, under the agenda of "last mile" and "leave no one behind", HFDBs are key for community health workers (CHWs) and mobile clinics in understanding the distance between health facilities in their areas to increase and optimize coverage of key interventions [2–4]. At the sub-national level, it is essential for the distribution of medical supplies and planning, while at the country level, it forms the basis for regulation, and public health functions and can also drive the development of new digital services by private companies. At the continent level, it holds the potential in promoting cross-border planning, pandemic preparedness, and collaboration.

World Health Organization’s (WHO) Geolocated Health Facilities Data (GHFD) initiative [5] outlines the value proposition of HFDBs as improving health system delivery, serving the population efficiently, and strengthening and supporting stakeholders in the health sector [6]. Specifically, it can be leveraged for decision-making, micro-planning (e.g., for vaccination campaigns), responding to health emergencies (precise maps of healthcare resources enabling faster responses [7]), and expanding primary care services in the context of universal health coverage (UHC). Visualization of health service provision capacities and their distribution can identify gaps [8, 9] and ensure equitable resource distribution to underserved populations. Stakeholders involved in resource allocation such as donors, and implementing organizations (e.g., distribution of bed nets [10]) will require a complete HFDB to increase efficiency gains compared to countries with duplicate or inaccurate facility lists [11]. A complete HFDB is key for interoperability with other users/sectors and systems while at the same time providing baseline data for reference and use in health research, analysis, and innovation [6].

Current population health needs and priorities of governments and regional entities in sub-Saharan Africa (SSA) require an HFDB with information on service availability and capacities. HFDBs form the foundation of disease surveillance systems, whereby data on cases diagnosed in facilities are continuously collected and aggregated through a routine health information system to inform decisions on public health measures [12]. During surveillance activities, HFDBs provide facility-level data for prioritization based on service levels and the population size of the catchment. In the context of WHO African region (AFRO), HFDBs are at the center of immunization activities and strengthening health systems. This includes planning for vaccine delivery, identifying types of health services provided at the facility level, and analyzing specific disease-prone areas to inform the allocation of medical supplies, human resources, and stock levels while addressing health service equity. Some of the governments in SSA, through DHIS2 (formerly District Health Information System Version 2), are already at the forefront in identifying their health facilities as a backbone of their health information systems [13].

The value of national and regional HFDBs across SSA became even more apparent during the planning and response to the Coronavirus Disease (COVID-19) pandemic [14–16]. Such SSA-wide HFDBs are critical for a coordinated health system response during pandemics and outbreaks. HFDBs are also essential for cross-national border coordination for outreach planning and optimization of regional immunization programs (e.g., synchronized polio supplementary immunization) [17, 18] and coordinated regional malaria control and elimination initiatives that involve facility-based data and/or service provision [19]. Further, HFDBs are key in managing healthcare access across borders for migrant populations and those residing in border areas [20, 21]. The 2019 and 2022 African regional geographic information system (GIS) summits emphasized the urgent need for updated HFDBs serving the WHO-AFRO [22].

Therefore, in the context of limited resources, a HFDB is a necessity; it should support, rather than compete with, health service delivery by making health systems more efficient. A good example is the experience of Haiti following the 2010 earthquake. At the time of the earthquake, a list containing only public-sector health facilities existed, and there was a lack of adequate information on the private sector (which provided major health services). Additionally, it lacked unique facility identifiers. A HFDB was created as part of the earthquake response [7]. The HFBD was instrumental in subsequent events, including a cholera outbreak, where the database was used to identify communities with no facilities which were targeted for setting up cholera treatment centers and units to provide care to the population in need. This demonstrates return on investment and how HFDBs can improve health outcomes through quicker response and reduced costs in case of subsequent emergencies. Such data-driven planning reduces redundancy and maximizes resource allocation efficiency.

While there is value and a strong business case for governments to create HFDBs, governments themselves also need to see value in them. For example, in Nigeria, the Federal Ministry of Health recognized the potential use cases of a HFDB through a consultative process. This process included the efficient management of facilities in the country by updating the status of a facility in the event of a new facility, a change in ownership, level, location, closure, change in accreditation status, or when a facility has multiple branches [23].

### What is the status of HFDBs in sub-Saharan African countries?

Despite the value of HFDBs, their availability remains a challenge for many SSA countries [24]. Most existing HFDBs contain suboptimal attributes (descriptors/variables for each facility) that only include administrative area, facility name, ownership, and facility type. They often only include public sector facilities [25], lack geographic coordinates, attributes of service readiness and service availability and facility capacity (e.g., the number of patients that a facility can attend to and number of beds), or are not regularly updated (i.e., provide only a cross-sectional snapshot). Importantly, periodic updates of HFDBs are often complicated due to the non-existence of a unique facility ID number [14, 25, 26], a recurrent issue that prevents unambiguous tabular joining with other facility-level data across health programs.

A number of factors could be attributed to the lack of HFDBs in most SSA countries. Key among these is the lack of political and financial commitment by governments. For example, developing a comprehensive facility list and maintaining it requires substantial investment (cost and time) especially in establishing the baseline list of facilities [27]. Further, non-appreciation of the value of geocoded data (business case) in decision-making which may be due to lack of expertise and resources to fully harness the utility of the HFDB. Lastly, inadequate infrastructure such as health information systems which are often not interoperable may also have contributed to the current state of HFDBs in SSA [28, 29]. Consequently, a SSA wide dataset has not been possible due to lack of the key prerequisite—country-level HFDBs—which are rare across SSA. Where HFDBs are available, countries, health agencies, and governments are understandably concerned about openly sharing data from HFDBs due to security and sometimes privacy concerns. While such concerns can be managed by outlining strategies for data sharing, such as anonymization and controlled access protocols, it is an issue that needs an honest conversation between stakeholders.

In this article, we advocate for a harmonized SSA-wide HFDB founded on country-level HFDBs. To achieve this, we elaborate on the steps required and challenges to overcome. We start by providing an overview of the minimum attributes of a HFDB and discuss past and current efforts to collate HFDBs at the country and regional (SSA) levels. Throughout this article, we argue that a HFDB should (i) include geographic coordinates of facilities and their corresponding subnational administrative unit, (ii) contain attributes of service availability and capacity, (iii) include facilities from both public and private sectors, (iv) be updated regularly, (iv) be available to health system stakeholders, populations and researchers through a data governance policy, and (v) issued by a national authority with an official mandate. We provide historical and recent examples to illustrate our points. We conclude by looking at key issues and challenges, such as privacy, legitimacy, resources, and leadership, which need to be considered to achieve complete HFDBs.

## Attributes of a HFDB

There is a set of minimum essential attributes for HFDBs which should be prioritized to maximize their usefulness on local, national, and regional levels. These attributes include facility name, unique ID, type, ownership, operational status, and, importantly, geographical coordinates with the relevant subnational unit (Table 1). Furthermore, countries developing their HFDBs should consider additional attributes through technical consensus with stakeholders to meet their specific needs. Several resources can be utilized, such as the GHFD initiative, which includes four unique identifiers, allowing countries the flexibility to add other data elements [5]. The Health Facility Assessment Technical Working Group led by United States Agency for International Development (USAID) proposed eight domains for core indicators to identify a health facility [28] which was adopted by Haitian HFDB [7]. Further, the International Hospital Federation, in collaboration with healthsites.io, defined 15 core attributes/identifiers [30]. With input from Médecins Sans Frontières (MSF) International, CartONG, and the Humanitarian OpenStreetMap Team (HOT), this foundational dataset was then mapped to OpenStreetMap[31], establishing healthsites.io as a Digital Public Good [32]. For a country without an official HFDB, initial efforts should be made to compile an openly licensed HFDB with unique IDs used across all in-country programs. This can be achieved by triangulating all existing independent lists within the country to derive a single list, as was recently done by a group of researchers and staff from different governmental agencies in Senegal [27]. Efforts such as the healthsites.io open campaign provide complementary approaches through on-ground validation exercises [30, 33]. The minimum essential attributes should be updated frequently and expanded to include private facilities. In later sections, we list possible sources to inform the initial effort of compiling a HFDB.

**Table 1:** Minimum attributes within a health facility database (HFDB). Numbers 1 to 7 and 8 to 12 show attributes that should be prioritized in the first and second stages of developing a HFDB, respectively

ID	Attribute	Description and/or examples
**1**	Name	Full name of the facility
**2**	Code	A unique code to identify a facility over time and ensure linkage to other lists
**3**	Subnational administrative region	The subnational administrative unit or a health district where a facility is located
**4**	Geographic coordinates	Geographic coordinates showing the precise location of a facility which can be used to update subnational administrative unit
**5**	Type	The type of a facility (primary to tertiary level)
**6**	Ownership	Public, private, faith-based organization, non-governmental organizations
**7**	Operational status	Operational or closed
**8**	Services	Type of services (e.g., emergency obstetric or cancer care)
**9**	Capacity	For example, number of beds, staffing and operating theaters
**10**	Infrastructure	For example, availability of water and electricity at the facility
**11**	Contact information	Physical address, and email address to reach the facility
**12**	Temporal data	Date opened and closed (if applicable), date of last update

Establishing a geocoded HFDB is merely an initial step. Equally important, a robust health system should be able to build on these minimum essential variables and include attributes of service availability and capacity linked with unique facility IDs. Therefore, all other program lists held by various in country programs, such as the immunization program, should reference the HFDB for essential attributes using the unique ID while at the same time feeding back to HFDB for updates of service and capacity. The ideal approach to compile a HFDB is a comprehensive census that records all essential attributes, services, and capacities of existing health facilities. For instance, in 2023, Kenya conducted a census incorporating both public and private facilities, collecting geographic coordinates, and adding data on health services and capacities while making the data open accessible [34]. In the next section, we outline the historical developments in forming geocoded facility lists and further explore the proposed minimum attributes.

### Geographical coordinates of facilities

#### Importance of geolocation in facility list

John Snow’s map of cholera cases and water sources showing that the distribution of the disease had a geospatial pattern related to drinking water sources in 1854 is a classic example of the utility of applying spatial dimensions to health data [35]. The value of geocoded facility lists to health service provision is vast. They facilitate the linkage between the geographical location of health facilities with disease events within the catchment populations they serve [36], across the urbanicity continuum (core urban areas to rural areas) [37–39], for example, computing malaria incidence using routine data [40] or estimating the number of children likely to use a health facility after an episode of fever or different health outcomes for programmatic planning [41]. This leads to a granular understanding of gaps in geographical accessibility to healthcare and deriving spatial patterns that can feed into national policymaking in the era of UHC [38]. Consequently, the disease burden, social dynamics, and environmental factors that influence the health needs of particular catchment populations can be integrated to better adapt health services to meet the specific needs of those populations. This could facilitate more effective, equitable, and disease-specific healthcare delivery.

There are multiple examples of how geographical coordinates in a HFDB have been used in health programs. They have informed micro plan maps and facility catchments for improved targeting of populations needing health interventions, such as polio vaccination campaigns, reaching zero dose children, and monitoring the efficacy of COVID-19 routine vaccinations [36, 42]. This might include using locations of facilities as reference for coordination, orientation, and knowing where the vaccines are stored. Other examples include determining subsets of the population that are geographically marginalized to different types of care, improving outreach activities by determining the optimal areas for CHWs linked to each facility. Further, databases of facilities have been used as gazetteers when geocoding villages and settlements and finally improving health outcomes as the location of facilities facilitates a well-coordinated and sustainable delivery of interventions in the areas that they are needed most [36].

Likewise, there are many examples of how geographical coordinates in a HFDB have been used in research. A geocoded database of health facilities in 50 countries in SSA [25] was used to estimate travel time to facilities within areas at risk of viral hemorrhagic fevers [43]. Vulnerable populations that would benefit from new health facilities and reduced travel time were identified. Facilities in the vicinity of at-risk populations were also recognized for prioritization in their readiness capacity to detect, treat, and respond to emerging pathogens [43]. During COVID-19, the same SSA facility list [25] was used to map geographical access to health facilities to inform where additional resources such as makeshift hospitals or transport programs might be needed for adults aged ≥ 60 years [15]. A recent body of work targeting the optimization of geographical accessibility to emergency obstetric and newborn care (EmONC) facilities [44, 45] has led to the adoption of the first maternal health indicator using travel time to the nearest EmONC facility within the framework of the Ending Preventable Maternal Mortality (EPMM) initiative (i.e., EPMM target 4 indicator) [46]. Perhaps, the most urgent and timely use will be the spatial precision needed for planning and response to Mpox [47, 48] in the same manner as COVID-19, Ebola [16], and cholera [7].

#### Considerations for accurately geolocating health facilities

It is vital to assign precise and accurate location attributes (geographical coordinates) in defining the location of a health facility (geolocation or geocoding). The gold standard for geocoding is using the location of a global positioning system (GPS) through a standard operating procedure. A review of sources of coordinates in national HFDBs across SSA showed that GPS locations are only available for a subset of facilities, predominantly public hospitals [25, 49]. Locations for most other facility types are derived indirectly by digitizing paper maps or using proxy locations from other infrastructures such as schools, digital gazetteers (a list of geographic place names and their coordinates), and base maps such as Geonames, Google Maps, Bing Maps, and OpenStreetMap (OSM) [25].

As geospatial technologies evolved and developed in sophistication [50], geocoding sources have improved, leading to a shift from on-screen digitization (most rudimentary) to reasonably complete digital gazetteers and base maps. For example, the number and type of contributors to OSM have grown, including voluntary mapping communities, governmental, non-governmental, and humanitarian organizations, for example, emergency health mapping campaigns, national OSM chapters, and “The Missing Maps initiative” [33, 51]. This has contributed significantly to accurately geocoding health facilities, for example, in Kenya and Senegal [8, 27]. Further, opportunities exist to exploit recent advances in geocoding and value addition from HFDBs. For example, the Starlink satellite system offers high-speed internet, especially in isolated and remote areas, and can be leveraged in health services mapping or the use of artificial intelligence (AI) in predicting health service access, especially in urban areas affected by traffic congestion and extreme climatic events [52, 53].

The growth in geospatial technologies has been tremendous to the point that we can attribute disease events (cases, outbreaks) and health services (availability and provision) to a specific location [50]. However, the application of these technologies has been heterogeneous across world regions. In high-income countries, locations of health facilities and other essential services (e.g., pharmacies, opticians) are part of well-defined registers with location parameters such as post-codes and street addresses. However, many low- and middle-income countries (including those in SSA) do not have such well-defined addresses, including defined location addresses of facilities [54]. There are efforts to develop systems that overcome the limitations of postal addresses. For example, through the what3words project [55], which divides the world into a grid of 3-m squares, assigning each square a unique three-word combination. This method is useful in regions with limited address infrastructure, such as remote areas and informal settlements.

#### Maps of health facilities in sub-Saharan Africa

**Fig. 1:**
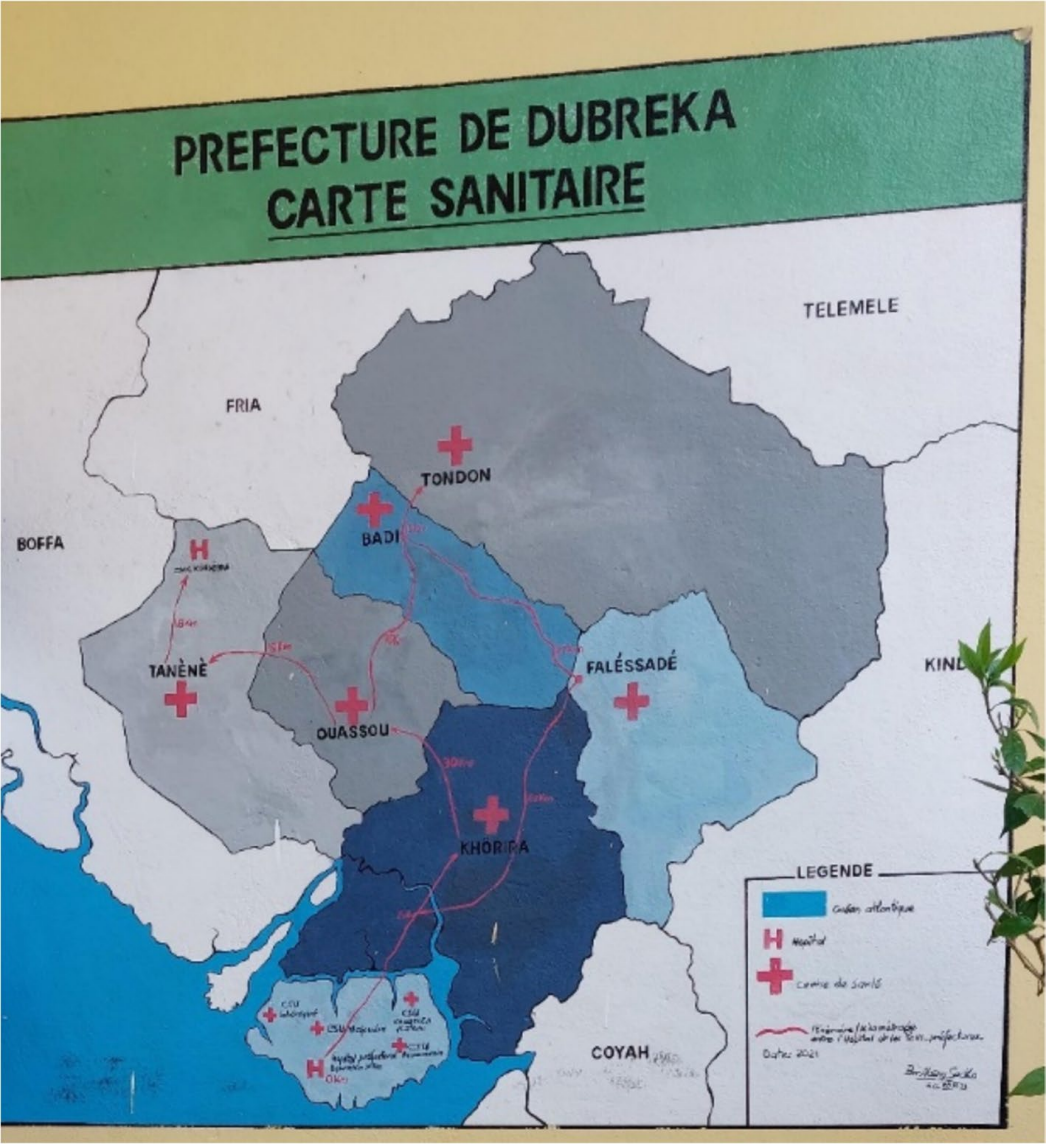
A wall map showing health facilities and subnational boundaries in Dubréka prefecture in Grand Conakry, Guinea in October 2024. (Source- authors image)

The use of geographic coordinates to make maps of health facilities is not new in SSA. During the colonial and immediate post-independence periods, SSA countries saw the value of displaying health facilities on maps. At the time, these were often hand-drawn maps of hospitals and health centers or incorporated as part of a country’s atlas [56]. For example, in the Democratic Republic of the Congo (DRC), the oldest available health service provider map archived at the WHO is dated 1953. In Kenya, mapping of facilities began before independence in the 1950s [56]. At the subnational level, district health offices displayed a map showing their health facilities, often hand-drawn or painted on a wall, a practice that has continued to this day (Fig. 1).

Over time, the practice of having a complete geocoded facility list represented on a map weakened. The lack of regular updates of facility lists that existed from the 1950s, coupled with an exponential, and in some places (predominantly urban areas), unregulated growth of the private sector, may have contributed to this situation. The lack of a single authoritative government-led HFDB has resulted in a situation where unofficial efforts/initiatives have had to be made to compile such data. While it does not necessarily follow that if a list is not official, it is of inferior quality; it is nonetheless likely to suffer from lack of data completeness, accuracy, precision, reliability, and not be openly licensed. Therefore, countries might be in positions with several conflicting and fragmented lists (with and without geographical coordinates) that were not officially endorsed by governments and often in portable document format, which made interoperability difficult [11, 57, 58]. These factors and the advances in geospatial science led to the gradual resurrection of geocoded lists of health facilities. Although some improvements have occurred to geocode locations of health facilities, the situation has not improved in the last decade [14]. Given the advancement of geospatial science the lack of progress is no longer due to technical issues, but due to a lack of financial resources needed to create and maintain HFDBs, political will, and coordination, which motivates our call for renewed attention. It presents the business and use case for an accurate and openly licensed baseline of health facility data.

### Typologies of facilities

Each country has a unique typology (a way of classifying the levels of facilities ranging from community health promotion, mobile clinics to tertiary referral hospitals) within the structure of the healthcare system, in both the public and private sector. Ideally, the categorization or levels of facilities should contain meaning relevant to the health system, such as ability to provide various services, ability to receive referrals, responsibilities for lower-level facilities, and numbers and cadres of health workers needed [59]. Therefore, two variables will be needed across HFDBs: a local (country-specific) typology of facilities reflecting the availability of services or levels of specialization and a harmonized typology (adaptable to accommodate these variations) across countries that allows comparison and cross-country analysis and planning. This inclusivity will enhance the comprehensiveness and relevance of the database across different contexts.

### Comprehensiveness of private and public sector

HFDBs, when originating from national authorities, have, by default, included public health facilities. However, completeness has been biased toward higher-level facilities such as hospitals. In creating a HFDB, the first step should be to adequately capture higher-level facilities in terms of location and services offered in both the public and private sectors. On the other hand, the care providers at the lower level, including the primary level, CHWs, pharmacies, and outreach centers, both formal and informal, should not be forgotten. An improved understanding of the number and distribution of such providers will have major implications for service delivery and optimization. This is important as countries balance service coverage through a mixture of fixed and outreach sites [60]. A recent call and guideline to implement a HFDB for CHWs have been made available [61] as a step in the right direction. For example, through the use of geospatial modelling, optimization studies in the deployment of CHWs in Sierra Leone [3], Mali [2], and Madagascar [4] have been conducted.

Despite their contribution to essential health service provision [62], private sector facilities have often been excluded from or underrepresented in HFDBs. Private facilities are a significant part of the healthcare system in SSA. In Benin (15%), Cameroon (24%), Congo (15%), DRC (16%), Eswatini (30%), Kenya (16%), and Uganda (17%), at least 15% of women gave birth in private sector facilities based on the most recent Demographic and Health Surveys (DHS) [63]. These proportions were even higher (> 30%) for care or treatment-seeking for children with fever in the private sector in Benin, DRC, Gabon, The Gambia, Kenya, Liberia, Nigeria, Tanzania, and Uganda [63]. However, the private sector is often not integrated, regulated, or accountable to the health system, with poor reporting rates in the routine health information system [64]. Most private facilities are in urban areas, and their operation location often changes, with a high turnover of closing and opening of new facilities. Private facilities are heterogeneous in size, services they offer, profit motives, and quality of care [65, 66]. This may explain why regulating and including the private sector in HFDBs is challenging.

While it is the role and mandate of the government to regulate private facilities, there are additional incentives in having private facilities as part of the HFDB. A substantial proportion of the population seek care from private facilities [63], the government contracts private facilities to provide healthcare and decongest the public sector[64], reimburses using health insurance-based finances [67], and distributes medicines, supplies and equipment such as bed nets to and through private facilities [64, 68]. For such applications, governments need to know where private facilities are located and the services they provide. Therefore, we call for facilities in the private sector to be included in national HFDBs. This would also enable future policies to improve their integration in health systems [65, 66, 69]. The attribute of ownership would specify the different sub-categories of private facilities, such as those operated by individuals, faith-based organizations (FBOs), non-governmental organizations (NGOs), for-profit companies, and insurance-based schemes.

### Services and capacity offered

South and colleagues posit that facility lists are of limited use if the services provided by facilities are not captured within a HFDB [14]. We view the expansion of HFDB attributes regarding services and the capacity of facilities as the second stage in developing a comprehensive HFDB (Table 1). However, a cross-sectional review conducted during the COVID-19 pandemic in SSA showed that service attributes were only available in the Kenyan HFDB [14]. Few countries have accurate, up-to-date information on health capacity or readiness to provide quality services despite decades of investments in health information systems [24]. Incorporating service availability and capacity at of each facility will support health service delivery and planning across different health domains, for example, assessing health accessibility and marginalization with respect to different types of services such as routine care [8], specialized and emergency care [49, 70–72], diagnostics, and mapping vulnerability, and risk preparedness for emerging pathogens [43].

Significant benefits for healthcare planning could have been realized if data on facility services and capacities had been available across SSA countries during the COVID-19 pandemic. For instance, these data could have enabled precise assessments of surge capacity for hospital and intensive care unit (ICU) beds and geographic access to critical care, providing actionable insights for policymakers and stakeholders [73]. Data on oxygen availability or the number of hospital beds or doctors could have informed the planning and response [14, 73, 74]. There are examples of studies assessing capacities and access to surgery and EmONC [49, 70–72] but less on other critical elements of service planning, including hospital care and diagnostics, due to a lack of data on services and capacity.

### Catchment areas and population

Assuming that an accurate HFDB is in place, a major challenge in resource planning and allocation is determining an accurate catchment population. This creates two challenges. First, a high-resolution and accurate population denominator disaggregated by age and sex is needed. Second, accurate and robust health facility catchment areas are needed. We address both of these elements in turn.

There is a need to maintain population data alongside a HFDB in order to ensure that the systems and service delivery are meeting the needs of its populations. In most SSA countries, population censuses are conducted every decade. In some cases, the most recent censuses were conducted over 30 years ago, such as in the Democratic Republic of the Congo and Somalia. The census data are not only coarse in the temporal domain but are usually also aggregated into coarse subnational units such as districts, counties, wards, or local government areas in the spatial domain. The long repeat period and low spatial resolution mean census data are inadequate to determine the catchment population at the health facility level. To this end, there a number of modelling initiatives that take in census data and range of covariates to disaggregate administrative level data to fine-scale (raster cell level or gridded level) estimates using spatial statistical approaches (machine learning, areal weighting, or dasymmetric approaches) [75, 76]. These approaches further disaggregate the estimates by age and sex while also projecting the estimates based on population growth rates. Such initiatives include Worldpop, Gridded Population of the World (GPW), High-Resolution Settlement Layer (HRSL), Landscan Global Population Database, Global Human Settlement Layer–Population (GHS-POP), and History Database of the Global Environment (HYDE) [75–77].

With all these high-resolution population datasets, perhaps the most challenging aspect for the end user is the lack of understanding which data product to use and when. The decision is likely linked to the quality of input population census, ancillary data, and the approach used for redistribution. A number of studies have been undertaken to compare these datasets systematically and can be used to inform choices made when HFDBs are being used together with population datasets to determine catchment populations [75–77]. Finally, it is worth noting that none of these population disaggregation initiatives are based in SSA, where the datasets are required most. This is a good example of what happens when governments do not own, collate, update, or publish their data. Consequently, modelling groups will step up and find a solution. However, this leads to a situation where SSA governments, institutions, planners, and researchers rely on that modelled data instead of real ground information. While the modelling fills in for the gap in data, it also absolves governments of the responsibility to collect and provide its own data.

Even when geolocated health facilities and population data are available in the required format, determining the catchment area—a geographical area delineated around a health facility describing the population that uses its services—has remained a challenge [37, 38]. Due to inaccurate catchments, facility-level estimates such as immunization coverage sometimes exceed 100%. A range of simple to complex approaches can be employed to define catchment areas, including buffers, and Thiessen polygons or based on modelled travel /distance or use of advanced spatial statistical models [37, 38]. The choice of either of these approaches is based on the availability of data especially geocoded data on the residential addresses of those seeking care and related care-seeking behavior. We urge countries to create, maintain and updated health catchment areas in the same breath as population and HFDBs. These will provide the foundation for the computation of population denominators for applications such as surveillance and for hard-to-reach populations.

### Updating HFDBs

Many existing country-level HFDBs lack a temporal dimension which allows changes in the capability, functionality, facility type (such as upgrades or facility designation), and attributes of facilities to be captured [25, 27]. However, we argue that a HFDB should be a living database and should, therefore, be updated and validated continuously. The mechanism for tracking changes in health facilities, such as closures, openings, relocations, allocation to administrative and health zones, and changes in capacity and facility type, is critical. This is particularly relevant for facilities outside the public sector, where some of these changes may be more frequent and less regulated. The HFDB resource package by WHO guides countries on various aspects that should be considered when updating a HFDB and how often it should be updated depending on a country’s local context [1].

Countries can align periodic updates of the HFDB with other regular activities, such as the delivery of medical supplies. Other possible avenues to take advantage to update facilities might include an annual re-accreditation system, where facilities are only re-accredited upon updating any annual changes [23] or through a DHIS2 module which prompts for updating any changes on an annual basis. The updates could also be mandatory through an act of parliament. For example, in Kenya, The Independent Electoral and Boundaries Commission is tasked to review the names and boundaries of constituencies at a predefined time interval in the Kenyan constitution.

**Fig. 2:**
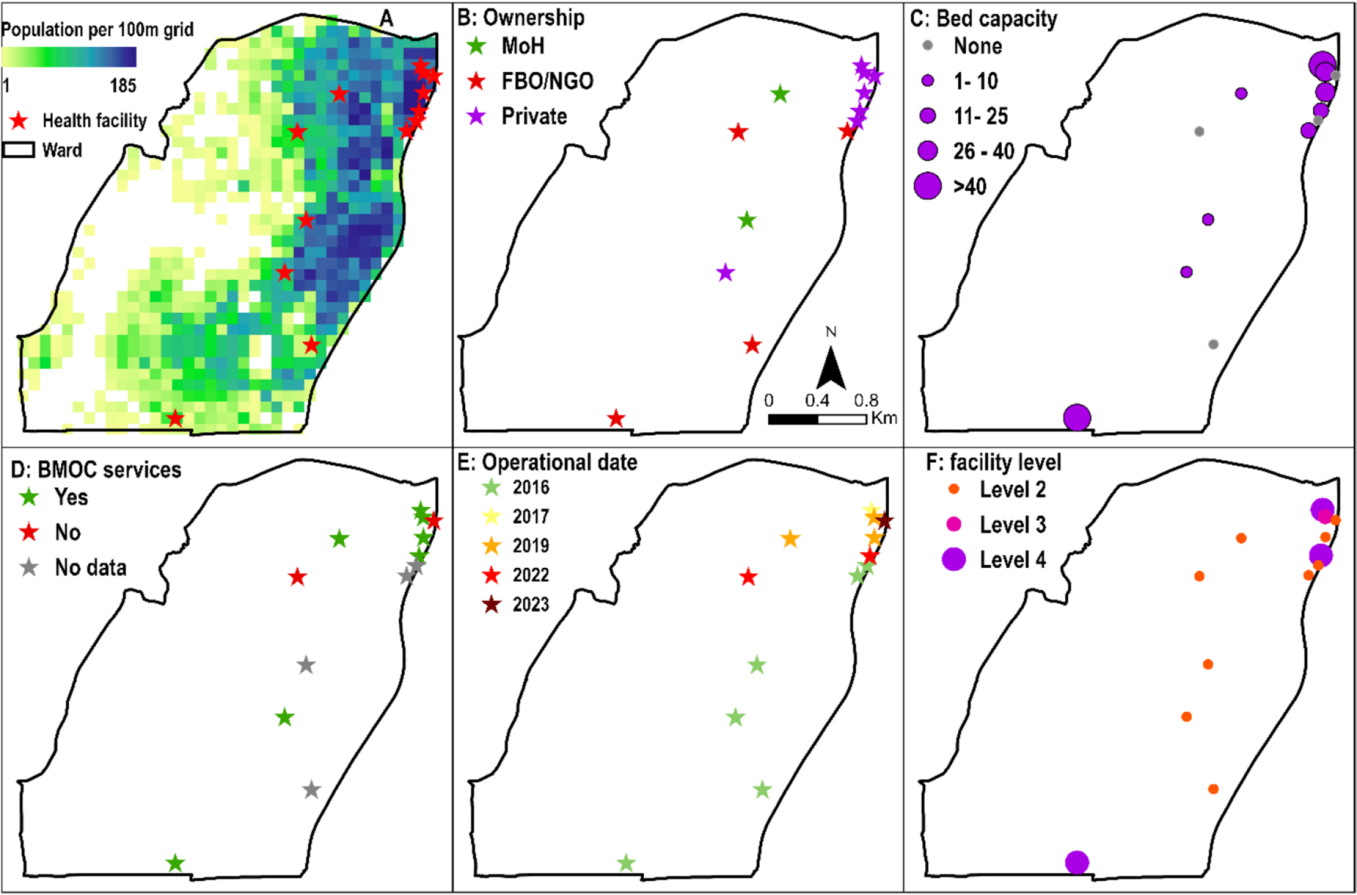
An illustration of the value of a comprehensive health facility database (HFDB). Data is based on openly accessible HFDB [34] for Bula Pesa ward (subnational unit) in Isiolo County, Kenya. Facilities with a population overlay show potential underserved areas (**A**). However, facility ownership (**B**), bed capacity (**C**), and higher-level facilities (**F**) are skewed towards private-for-profit facilities with implications on where the poorest live. Basic obstetric care (BMOC) facilities shown in **D**, key in the reduction of preventable maternal deaths. The date the facility was approved and became operational (**E**) shows that historically, many facilities were in the south and more recently private facilities opening in the northeast area

Understaffing due to resources in relation to updating HFDBs is often a limitation in the regular update of HFDBs, which are usually highly dynamic and politically sensitive. Staffing is not the only issue. The rate at which attributes of facilities—particularly operational status, services, and capacity—change can be high, especially in urban areas. For example, within a month, a private clinic might be closed by health authorities, another facility with the same name opens two streets down, and a pharmacy with a different name opens in the original location. This makes it resource intensive to maintain an up-to-date national HFDB. Overall, the value of essential minimum attributes of a HFDB area is illustrated in Fig. 2.

## Building HFDBs in SSA

### Which initiatives are working on compiling HFDBs?

Several past and current initiatives have created lists of health facilities in SSA. These are varied and may involve harmonizing facility lists obtained from various sources [27] or crowdsourcing [78], as summarized in Table 2.

**Table 2:** A summary of programs/projects or initiatives harmonizing health facility lists (HFDB) in SSA countries. The table is ordered by UN and WHO led (accountable to member states) followed by project-based initiatives that are not directly affiliated with any government or member states

Contributor	Description (region, year, ownership, open access-OA, and geocoding)
WHO-AFRO	WHO-AFRO houses a static, open access, web-based HFDB of over 85,000 public health facilities across different years. Its attributes include ID, administrative unit, facility name, type, and ownership, available to the public [79]
GHFD	WHO, under its Geographic Information Systems (GIS) Centre for Health, motivated by filling the data gaps witnessed during the COVID-19 pandemic, launched an ambitious effort—GHFD—in March 2022 [5]. Through seven strategic pathways, the initiative seeks to provide support for developing a georeferenced HFDB per country that is maintained, actively used, and publicly shared by respective Ministries of Health (MoH). Countries through MoH will maintain and regularly update their HFDB; elements of these lists will then be shared as a global public good through the ministry’s website, which will be referenced in a global directory. It will include unique identifier, name, type, and location, but countries are flexible to add other data elements. The initiative has yet to provide the directory. Two and half years since it was launched, it remains a massive task if all member states are to be in a position to regularly update their HFDB by 2027 with all necessary mechanisms in place
Humanitarian Data Exchange-HDX	HDX is a global, open access data sharing platform by the United Nations Office for the Coordination of Humanitarian Affairs. It includes geocoded health facilities from various sources and specific countries [25, 80] covering different years
GeoPoDe	Like HDX, GeoPoDe is an open access web data repository for geospatial datasets available by country (21 in SSA) across different years including geocoded health facilities, now under the ownership of WHO. Data are available for non-profit or humanitarian applications [81]
Healthsites.io and OpenStreetMap (OSM)	The Global Healthsites Mapping Project is building a baseline of health facility data with OSM [30]. It is an open-source Digital Public Good with support for OSM and interoperability between information systems. It supports MoH in maintaining health facility data and enables multiple stakeholders to collaborate on a shared database [33]. It leads data validation through its Emergency Health Mapping Campaign by building trust between local communities, governments, and health authorities [78]. As of August 2024, it had 78,594 sites, including pharmacies in the public and private sectors in Africa. A small proportion have details about the capacity and services [14]. The data is available under an open data license and is accessible through healthstes.io, OpenStreetMap.org, and HDX
Google Maps	Like OSM, Google Maps relies on volunteered geographic information, in addition to publicly available or licensed third-party data, to provide a global open data, geocoded HFDB of public and private facilities across different years [82, 83]. It is updated and had 214,052 hospitals and medical centers globally, with 73,365 in low and lower middle-income countries in 2022 [83]. In 2023, only 53% of facilities in healthsites.io were available in Google Maps across the globe, with most facilities mapped for higher-income countries [83]
GRID3	Since 2017, The Geo-referenced Infrastructure Demographic Data for Development (GRID3) has been helping governments in SSA make better use of spatial data in healthcare among other sectors [84]. Notably, it has facilitated assembly of open access, geolocated facility lists in the Democratic Republic of Congo, Nigeria, Zambia, and the Sierra Leone. However, these lists contain minimal attributes of service availability and capacity. In 2021, GRID3 is one of the organizations supporting the WHO with the coordination and implementation of the GHFD initiative in the Africa Region
KEMRI-WTRP	The Kenya Medical Research Institute-Wellcome Trust Research Programme (KEMRI-WTRP) compiled a pioneering but static (2012–2018) database of 98,745 public facilities [25] in 50 SSA countries. The list was made open data to incentivize countries and stakeholders to update and further validate the list [25]. The list has been utilized widely by researchers and stakeholders across the globe for research (cited over 200 times in Google Scholar) and implementation in SSA [25]. It is yet to be updated 6 years after its publication [14, 80]

### Which countries have openly accessible HFDBs?

Comprehensive national HFDBs which are openly accessible are still rare in SSA countries [14, 26, 80, 82]. In 2020, only 7 of the 52 SSA countries had an open data, downloadable, geocoded health facility list (Kenya, Malawi, Namibia, Rwanda, South Sudan, Tanzania, and Zambia), accounting for only 16% of the SSA population [14]. Additional file 1: Table 1 shows 29 countries which, as of October 2024, published a form of HFDB on the internet. The list of facilities can be downloaded as a file (geospatial, portable document file) or a table with a range of attributes.

## Looking ahead

There are important opportunities and challenges which should be considered and addressed within the space of creating HFDBs nationally and harmonizing them regionally. These pertain to (i) opportunities to improve HFDB, (ii) stewardship and leadership, (iii) partnerships, (iv) whether HFDB data should be made open access, and (v) whether it is valuable to have a harmonized regional, SSA-wide database. We discuss each in turn.

### What are the opportunities to improve HFDBs?

There are several limitations to current initiatives and efforts (Table 2 and Additional file 1: Table 1). One of the main limitations with OSM, Google Maps, and similar services is that beyond geographical coordinates (often not validated/officially endorsed), these platforms have extremely limited data on linkable unique facility IDs, service type, ownership, and capability. While there are examples of how such problems can be addressed through collaboration with health authorities [33] and inclusion of a unique ID as done in healthsites.io, some opportunistic platforms can be harnessed, as summarized in Table 3.

**Table 3:** Platforms that can be harnessed by SSA governments to create and update HFDBs

Platform	Description
***Routine health information***	Systems have a huge potential in supporting development of HFDBs. Forty-three SSA countries have implemented a national health information management system based on DHIS2, a web-based open-source platform [13] that provides a significant transformation in health information in SSA. For a country to set-up a DHIS2, it will obviously require a form of a HFDB. Facilities will then make monthly reports, which can be used to infer their capacities and services, with some having their geographical locations mapped, information that can feed back to the HFDB. The DHIS2 list should theoretically be the same as the country’s HFDB, but a previous audit in Kenya showed a discrepancy of 17% [8]. As governments have implemented DHIS2 over time, this information can be harnessed to understand the temporal changes in facility levels and operational status. However, reporting completeness and data quality are still poor. For example, some of the unique IDs do not match between DHIS2 and HFDBs in some countries, though progress is being made [8, 64, 85, 86]
***The Polio Eradication Programme***	In the WHO African region has implemented strategies to strengthen surveillance, routine immunization, and monitoring and evaluation. One strategy uses open-source data collection tools for integrated supportive supervision and electronic surveillance visits to healthcare facilities [26]. To facilitate this process, countries submit a list of health facilities (45 of the 47 member states) which are often incomplete with no GPS coordinates. During visits to facilities, data on facility name, their locations and routine immunization capabilities are collected and used to develop, update, and validate HFDB for the WHO African region countries [26]. This initiative started in 2017 with the inauguration of the AFRO-GIS Centre to support innovative technologies for Polio surveillance [26]
Through the ***health resources and services availability monitoring system (HeRAMS)***	The WHO facilitates a collaborative process through which health service providers exchange, analyze, and validate information on essential health resources and services. This includes the compilation, maintenance, update, and dissemination of an authoritative HFDB. As of June 2024, HeRAMS was available in nine SSA countries, with two additional countries in a preparatory phase [87]. It shows geolocated health facilities with attributes including service offered, e.g., 1241 facilities in DRC and 8077 facilities (including community health workers) in Mali [88]. The system offers a mechanism to produce, maintain, and validate a HFDB. However, access and use of facility-based indicators in the HeRAMS database are dependent on ad hoc permission by service providers engaged in the process at the national level. Once access is granted, the data can also be prepared via an R package for subsequent geospatial analysis to identify the populations that are geographically marginalized [89]
***The Service Provision Assessment (SPA)***	Is a health facility survey that collects data on service availability within a country’s health system. While the sample size is about 400–700 (or a census of facilities, depending on the total number of facilities and funding available), SPAs can be used to update some aspects of HFDB. Between 2004 and 2022, 17 SPA surveys have been conducted in 10 countries. With seven annual SPA surveys between 2012 and 2017, Senegal used SPA data to in curate its HFDB [27]. The SPA, together with WHO’s service availability and readiness assessment (SARA) and the World Bank service delivery indicators are now part of the harmonized health facility assessment [24]
***Health Electrification and Telecommunications Alliance (HETA)***	Is a USAID, Global Development Alliance aiming to reach health facilities across the region for electrification. Through HETA, facility attributes can be updated in HFDBs [90]
***Health insurance companies***	In SSA countries often hold a very detailed list of facilities with whom they have contracts. Such lists can be harnessed to improve HFDBs [91]

### Stewardship and leadership

The ultimate obligation and role to set up, develop, and update HFDBs resides with a country’s MoH. The MoH is the leading curator, user, and owner of the HFDB for healthcare planning and provision to the population. For HFDBs to be sustainable, governance structures should be formalized through program-specific policies, standard operating procedures, specification of metadata, and making them accessible. This will prevent loss of information and skills, for example, from staff turnover.

Further, sustainability of the HFDBs will depend on financial and political commitments from the national governments in SSA. Indeed, domestic financing and investments in national health data infrastructure including HFDBs is essential. Recent changes in global health and development financing where USAID was paused and or terminated for some programs has important consequences on the health outcomes including health data especially in SSA [92]. For example, the DHS Program, co-financed by USAID has been collecting reliable data on health and demographics in over 90 LMICs, was halted [93]. While reductions in external financial support should be gradual for ensuring data continuity, the ‘project-based’ mindset of funding individual programs and/or entities to develop their own facility lists is unsustainable and may have led to duplicated efforts. Although in the short-term, the acute discontinuity of funding leaves a data vacuum behind, it can be used as a stepping stone (opportunity) by countries and regional entities to strengthen ownership and oversight of their health data on the long term. At the same time, reimagining a system less dependent on external funding to one that is anchored in domestic financing and investments.

When the utility and value of these lists are harnessed by governments and made accessible to civil society and broad health sector stakeholders, it will enhance future updating of data and a meaningful dialogue of gaps. Data reuse will facilitate updates and maintain data quality [94]. When the MoH is at the center of creating a country’s HFDB, it ensures efforts are not made in parallel but through coordination that builds upon existing work for sustainability. For example, between 2010 and 2016, six government departments and agencies partnered with different development organizations to create ten health facility and service availability lists in Nigeria [11]. It is likely that across countries and within countries in SSA (for example, by health service area or disease program), such duplicated lists exist as seen from countries that harmonized their HFBDs [27, 57, 69]. On the other hand, Kenya is a well-documented case study of how to develop one single geocoded HFDB between the early 2000s and 2021 [8, 57, 64, 69]. A culmination of these efforts was a census of all 14,883 health facilities in Kenya in 2023 [34], which may provide a benchmark for countries without HFDBs in creating a baseline. The MoH led the census and was supported by government agencies, county governments, development partners, the private sector, and other stakeholders.

### Partnerships

While some MoHs create HFDBs independently, others collaborate with national partners to build the data repositories, for example, in Kenya [8, 34, 57, 64, 69] and Senegal [27, 33]. An enabling and open environment allows citizens and stakeholders to support MoHs in improving baseline health facility data. Many actors in the health sector operate nationally and internationally and contribute to building country-level HFDBs together with MoHs. This includes international bodies (WHO, UNICEF, UNFPA), donors, implementing and humanitarian partners (The International Rescue Committee (IRC), national Red Cross/Red Crescent societies, MSF, and Population Services International), technical advisors, and research institutions. These stakeholders often already have independent, project-based facility lists, often at sub-national level. However, we argue that every partner should use the central MoH-held HFDB and feed back to it any changes made during a project, through a well-defined centralized update mechanism. For example, on-site visits and data collection efforts of partners should support validation and updating of GPS coordinates and other attributes in the MoH-held HFDB.

### Should HFDBs be open data?

Whether and how frequently the MoH should make HFDBs (or parts of the list) available to stakeholders and researchers rests on the respective country. It is often a common view (also held by many governments/MoH) that geocoded health facility data should be confidential. Countries in SSA barely share even basic health facility data with in-country partners (such as NGOs), often citing sensitivity issues. The sensitivity of sharing national data across countries within a region such as SSA warrants further interrogation and a regulatory framework to facilitate data sharing as a common resource for public health purposes. This may involve a governance framework that balances data sharing with rules for protecting the interests of data subjects, creators, and sustainability [95]. There needs to be a much broader dialogue, across communities of practice, governments, and international agencies especially on ethics in the use of geocoded geospatial data in general and particularly HFDBs [96]. The WHO advocates for sharing baseline data under an open license.

HFDB can be viewed as having two categories of data. The first category is information that stakeholders, the public, and patients find useful. This may include information such as the location of emergency rooms, dentists, medical laboratories, and some of the key attributes as is done in high-income countries [97]. Making this information available will drive the development of online services (by public or private actors) to benefit and inform the population about service availability (and other characteristics, e.g., service quality, including through client reviews). For the purposes of accountability and governance, these types of data should be public, ideally available from a government website and updated for health users, facilities, government, and researchers. When the MoH shares HFDBs (or parts of HFDBs) through well-laid-down structures and policy environments, this may lead to improvements in data quality, interoperability, and a potential source of revenue internationally [1].

The second category entails types of data that will not be included in the openly licensed HFDB, due to sensitivity (known as “service endorsed” component), and are classically kept by dedicated national health programs and not shared publicly. This maybe also include information linked to services with very rapid changes. In that case, the specific health programs in the countries should maintain that information, but with a clear “table joint” (unique identifier to link databases) with the HFDB so that it is easy to map and analyze nationally whenever needed. That is, the availability of services and capacity may often be sensitive; for example, data on laboratories handling infectious materials is not typically shared by countries. In countries with such kind of service endorsed data, current efforts should be directed to rapidly implementing and sharing an MFL with all the other components. This would immediately provide the benefits associated with HFDBs [6] while service availability information can be obtained through a memorandum of understanding (MoU) with specific health programs.

In general, data sharing is an overarching issue which is governed by national data protection acts, data governance, and legislation which sometimes prevents sharing some datasets. The broad health data sharing issues within and between countries remains a major limitation to cross-border, regional data use efforts. In SSA contexts, for example in Uganda, lack of resources, poor data quality, restrictions, leadership, and inter-organizational boundaries hinder health organizations from sharing geospatial data [98]. Many examples exist where national health data are not shared, for example, scrambling of geographic coordinates in the DHS data before sharing them publicly [99]. On the other hand, the coordinates of health facilities collected within the Service Provision Assessment (SPA) are not scrambled. This has been useful in creating some HFDBs (for example, in Senegal [27]). However, it remains a challenge since most SPAs often sample a subset of facilities within a country and are thus not comprehensive (e.g., defining health facility catchment at country level would be problematic). Nevertheless, this is a good example of controlled data access that can be replicated and adapted within the country context to facilitate the sharing of HFDBs.

Overall, such issues linked to the sharing of SSA-wide HFDBs can only be resolved by an agency such as WHO or UN as the oversight agency to facilitate agreements or a charter from member states. The agreement or the charter would outline the sharing of minimum data on health service provision (facility name, geographic coordinates, and facility ID). The alternative (counterfactual) might include either an external agency/company compiling the data and making it open access to all member states [25, 49] or "fuzzification" of locations to facilitate sharing HFDBs as open data.

### Continental/regional (SSA) wide HFDB

A continent-wide database is a global public good that will lead to innovation [5] and improved health. It spans management, planning, and coordinating response to disasters, outbreaks, and pandemics such as COVID-19 [14–16]. Countries are already conducting synchronized polio supplementary immunization activities across national borders in western and central Africa [18] and some parts of eastern Africa [17], which improves planning, coordination, and optimization of regional immunization activities. HFDBs are also key in the control and elimination of malaria across borders. For example, countries in the Southern African Development Community Malaria Elimination Eight (Botswana, Namibia, South Africa, Eswatini, Angola, Mozambique, Zambia, and Zimbabwe) established 39 border health posts across their international borders to enhance control malaria particularly for mobile and migrant populations [19]. Indeed, the users of facilities know no boundaries when seeking care. For instance, Ssengooba and colleagues demonstrated that across the national border sites of Uganda, Kenya, and Rwanda, about one-third of the border population sought care across the border [21]. Therefore, there is a need for closer cooperation, policy, and legal framework to realize health synergies for these communities; HFDBs will be useful in such cross-border initiatives [20, 21].

The first pioneering SSA HFDB [25] (described in Table 2) was possible because it was a research product led by a research institution. Although this database served the public health community well, the question arises regarding who should manage such a continental-level HFDB—it has not been updated since its publication 6 years ago. We also recognize that other challenges must be addressed before updated country-level HFDBs become ubiquitous. On the positive side, since the publication of the first SSA database, many developments have occurred; geocoding methods have improved, more lists have become available, and additional actors (data providers) have emerged. An updated version of this list will facilitate updated spatial metrics in SSA [15, 43, 49, 70, 82, 83] and indicators of service availability and capacity are included, for which we strongly advocate.

The GHFD initiative (described in Table 2) is a welcome endeavor which has the potential to support countries and mobilize relevant stakeholders [5] for an SSA HFDB. The GHFD proposes this as a global good that can be harnessed with geospatial analysis. While it is the role of WHO to support countries, data sharing is not their role. Whether we succeed at creating a continental HFDB depends on countries and on harmonization efforts under the GHFD initiative and to what extent they can bring other actors on board [14, 80, 83, 100]. However, we argue that some agencies are less constrained and can share and consolidate HFDBs in SSA. To harmonize such an SSA-wide HFBD database, the current UN initiatives (e.g., UN OCHA-HDX) would be better positioned to lead as an endeavor. To garner trust and legitimacy among governments and members, an SSA-based entity such as WHO AFRO, or Africa CDC would need to host such an initiative. 

## Conclusions

The significance and value of a temporal, geocoded open HFDB with attribute data on facility capacity and services to SSA countries cannot be overstated. There is huge value for governments and funders to invest in a harmonized, geolocated HFDB for their respective countries. There are many unique use cases. This includes efficient service delivery (e.g., distribution of medical supplies), the regulation of facilities and public health functions, planning for health services, disease surveillance, and reaching zero-dose children for vaccination. Individuals will directly benefit from knowing where facilities are located, what services they offer, and, more importantly, having a choice (preference) while communities can keep health facilities accountable and facilitate optimization of community outreach programs. The efficiency will lead to cost savings (strengthening healthcare systems towards UHC). HFDBs can also drive the development of new digital services from private companies. Therefore, we call all actors and stakeholders to join and support countries in their HFDBs journeys. When each country collates, harmonizes, maintains, and updates its HFDB and makes this open access, harmonization to an SSA-wide HFDB is achievable. A SSA-wide HFDB will support pandemic and epidemic preparedness, including surveillance for diseases such as Ebola and COVID-19. It will enable targeted emergency actions and appropriate responses to emerging pathogens like Mpox, promote and facilitate cross-border disease control and elimination, and plan efficient service delivery to border populations, including immunization outreach and malaria elimination.

## Supplementary Information

Additional file 1. A table showing country-specific health facility databases in sub-Saharan Africa

## Data Availability

No datasets were generated or analysed during the current study.

## Abbreviations

AFRO: The World Health Organization regional office for Africa
AI: Artificial intelligence
COVID-19: Coronavirus Disease-2019
DHIS2: District Health Information Software 2
DRC: Democratic Republic of Congo
EmONC: Emergency obstetric and newborn care
EPMM: Ending Preventable Maternal Mortality
FBO: Faith-based organization
GHFD: Geolocated Health Facilities Data initiative
GRID3: The Geo-referenced Infrastructure Demographic Data for Development
GPS: Global positioning system
GIS: Geographic Information Systems
HFDB: Health facility Database
HDX: Humanitarian Data Exchange
HeRAMS: Health Resources and Services Availability Monitoring System
HETA: Health Electrification and Telecommunications Alliance
ICU: Intensive care units
ID: Identification
KEMRI-WTRP: The Kenya Medical research institute-Wellcome Trust Research Programme
MFL: Master health facility list
MSF: Médecins Sans Frontières
MoH: Ministry of Health
NGO: Non-governmental organization
OA: Open access
OSM: OpenStreetMap
SARA: Service availability and readiness assessment
SSA: Sub-Saharan Africa
SPA: Service Provision Assessment
UHC: Universal health coverage
UN: United Nations
UNICEF: United Nations Children's Fund
UNFPA: United Nations Population Fund
UN OCHA: United Nations Office for the Coordination of Humanitarian Affairs
USAID: United States Agency for International Development
WHO: World Health Organization
WHO-AFRO: The WHO regional office for Africa
