# Supplementary material for: Putting health facilities on the map: a renewed call to create geolocated, comprehensive, updated, openly licensed dataset of health facilities in sub-Saharan African countries
Source: BMC Med. 2025 Apr 7;23:211. doi: 10.1186/s12916-025-04023-z (PMC11978184; doi:10.1186/s12916-025-04023-z)
Supplement: Supplementary file 1 — Additional file 1. A table showing country-specific health facility databases in sub-Saharan Africa [file 12916_2025_4023_MOESM1_ESM.pdf]

Additional file 1

Additional file 1: Table 1 : Country-specific health facility databases in sub-Saharan Africa

Country	Accessible online	Data freely downloadable	Data format	Downloaded data geocoded	URL	Last updated	Owner	License	Additional details
Benin	Yes	Yes	Excel	Yes	https://dev.cartesanitaire-benin.org/data#section-info	No date	Ministry of health		Facility list with region, facility name, DHIS2 id and coordinates.
Botswana	Yes	yes	Excel, Pdf	Yes	https://healthfacilities.gov.bw/facilities	No date	Ministry of health		Facility list with district, facility name, ownership and coordinates.
Burkina Faso	No	Yes	Pdf	No	http://cns.bf/IMG/pdf/carte_sanitaire_2010.pdf	2010	Ministry of health		Report with maps and listing facilities in different regions.
	No	Yes	Pdf	No	https://www.afro.who.int/sites/default/files/2018-08/Profil%20sanitaire%20du%20Burkina%20%202.pdf	2017	Ministry of health		Report with a map on page 17 of health facilities.
Burundi	No	No	Web page	No	http://41.79.224.88:8080/jw/web/login		Ministry of health		Website with a map of health facilities
Cameroon	No	Yes	Pdf	No	https://dhis-minsante-cm.org/portal/index.html	No date	Ministry of health		Map of Cameroon showing the health facilities.
The Central African Republic	No	Yes	Pdf	No	https://www.sante.gouv.cf/sites/default/files/2022-04/cartedistrictssanitaires2018.pdf	No date			Map of health facilities
Chad	No	Yes	Pdf	No	https://reliefweb.int/report/chad/carte-sanitaire-du-tchad-2019-2023	2019	Ministry of health		A report with a map showing distribution of health facilities.
	Yes	Yes	Excel	Yes	https://data.humdata.org/dataset/chad-list-of-health-facilities-and-health-districts?	March 2021	OCHA		Facility list with facility name, region and coordinates
Democratic Republic of the Congo	Yes	Yes	Excel, Shapefile, csv, Geodatabase, KML, GeoPackage, SQLite Geodatabase	Yes	https://data.grid3.org/datasets/GRID3::grid3-cod-health-facilities-v1-0/about	May 2024	Center for International Earth Science Information Network (CIESIN), Columbia University and Ministère de la Santé Publique		A Facility list in different formats.
	No	No	Web page	No	https://dev.cartesanitairerdc.org/data	No date	Ministry of health		Website with map of health facilities.
Cote d'Ivoire	No	No	Web page	No	https://cartesanitaire.ci/	No date	Ministry of health		Website with map of health facilities
Ethiopia	No	no	Web page	No	https://mfrv2.moh.gov.et/#/dashboard	No date	Ministry of health		Website with list of facilities; name, type, operational status and region. No coordinates and requires login for download.
Eswatini	Yes	Yes	Excel	No	https://www.gov.sz/list%20of%20health%20facilities%20by%20region.xls	No date	Ministry of health		File listing facility names and regions
Guinea	No	No	Web page	No	https://portail.sante.gov.gn/carte-des-structures-de-sante-en-guinee/	No date	Ministry of health		Website with map of health facilities
Kenya	Yes	Yes			https://kmhfr.health.go.ke/	No date	Ministry of health		Requires login for download
Lesotho	Yes	No			http://www.chal.org.ls/hospitals.html	No date	Christian Health Association of Lesotho (CHAL)		Website listing health facilities
	Yes	No			http://www.chal.org.ls/healthcentres.html	No date	Christian Health Association of Lesotho (CHAL)		Website listing health facilities
Malawi	Yes	Yes	Excel, pdf	Yes	https://zipatala.health.gov.mw/facilities	No date	Ministry of health		Facility list in different formats
Mali	No	Yes	Pdf	No	http://www.sante.gov.ml/index.php/nep-mali/item/1558-carte-sanitaire-du-mali-mise-a-jour	2012	Ministry of health		Report with health facility maps
Mauritania	Yes	Yes	CSV, shapefile,	Yes	https://tanausu-pg.carto.com/datasets	No date			Facility lists in different formats

			KML, GeoJSON, GPKG						
Mauritius	No	Yes	Pdf	No	https://health.govmu.org/health/wp-content/uploads/2023/03/Health-Map-2016_Print.pdf	2015	Ministry of health		Map of health facilities
Mozambique	No	No	Webpage, XML	No	https://sis-ma.in/?page_id=740	2014	Ministry of health		Website describing the health structure and has an xml link to facility list
Namibia	Yes	Yes	Excel, pdf	Yes	https://mfl.mhss.gov.na/location-manager/locations	2018	Ministry of health		Facility lists in different formats
Niger	No	Yes	Pdf	No	http://library.procurementmonitor.org/backend/files/List%20of%20Coded%20Health%20Facilities%20in%20Niger%20State.pdf	No date			Listing facilities in different regions
	Yes	Yes	Excel	Yes	https://www.cartesanitaireniger.org/data	No date	Ministry of Public Health, Population and Social Affairs		Facility list with facility name, region and coordinates
Nigeria	Yes	Yes	Excel	Yes	https://hfr.health.gov.ng/about-us	No date	Ministry of health		Facility list is downloadable after signing up on the site
	Yes	Yes	Shapefile, csv, KML, GeoJSON	Yes	https://data.grid3.org/datasets/GRID3::grid3-nga-health-facilities-/about		CIESIN, Columbia University and Ministère de la Santé Publique	Free to use	Facility lists in different formats
Rwanda	No	Yes	Pdf	No	https://rwandabar.org.rw/attached_pdf/Medical%20facilities%20in%20Partnership%7C%20RBA-1621592808.pdf	No date			Listing of facilities in different locations
	No	No	Web page	No	https://rmdc.rw/spip.php?article11	No date	Rwanda medical & dental council		Website listing health facilities in different regions
	No	No		No	https://aggregate.moh.gov.rw/	No date	Ministry of health		Links to HMIS, requires logins
Senegal	Yes	Yes	Excel	Yes	https://doi.org/10.1038/s41597-024-02968-z	2023	Institut de Recherche en Santé de Surveillance Epidémiologique et de Formations (IRESSEF), Dakar, Senegal		Research article with github link to health facility list output of the work
	No	Yes	Pdf	No	https://www.esante.sn/app/uploads/repertoire-structures-de-sante-privees-senegal.pdf	No date			Pdf listing facilities in different locations.
South Africa	Yes	Yes	Excel	No	https://dd.dhmis.org/orgunits.html?file=NIDS%20Integrated&source=nids	No date	Department of health		List of facilities without coordinates.
Tanzania	No	No			https://hfrs.moh.go.tz/web/index.php	No date	Ministry of health		Download requires request to ministry of health.
Togo	No	Yes	Pdf	no	https://sante.gouv.tg/wp-content/uploads/2024/09/Rapport_Annuel_Performance_2020_DRSGL_14_04_2021_VF.pdf	2020	Ministry of Health		Pdf report with a map of health facilities in some regions of the Togo
Uganda	No	Yes	Pdf	No	http://library.health.go.ug/health-infrastructure/health-facility-inventory/national-health-facility-master-facility-list-2018	2018	Ministry of health		Pdf listing facilities in different locations.
Zambia	Yes	Yes	Excel	Yes	https://mfl.moh.gov.zm/facility/index	No date	Ministry of health		List of facilities with coordinates.

Footnote: Geolocated MFLs for Angola, Botswana, Cabo Verde, Comoros, Congo, Equatorial Guinea, Eritrea, Ethiopia, Gabon, The Gambia, Ghana, Guinea-Bissau, Liberia, Madagascar, Seychelles, Sierra Leone, South Sudan, and Zimbabwe can be shared on request from WHO/AFRO-GIS Centre (https://www.afro.who.int/gis-centre). Geographical coordinates have been validated for Gabon, Uganda, Malawi, Mozambique, Ethiopia, and Gambia.
